# Stacked Integration of MEMS on LSI

**DOI:** 10.3390/mi7080137

**Published:** 2016-08-05

**Authors:** Masayoshi Esashi, Shuji Tanaka

**Affiliations:** 1 Micro System Integration Center (μSIC), The World Premier International Research Center Advanced Institute for Materials Research (WPI-AIMR), Tohoku University, Sendai 980-0845, Japan; 2 Graduate School of Engineering, Tohoku University, Sendai 980-8579, Japan; tanaka@mems.mech.tohoku.ac.jp

**Keywords:** MEMS, LSI, stacked integration, wafer level transfer, through Si vias

## Abstract

Two stacked integration methods have been developed to enable advanced microsystems of microelectromechanical systems (MEMS) on large scale integration (LSI). One is a wafer level transfer of MEMS fabricated on a carrier wafer to a LSI wafer. The other is the use of electrical interconnections using through-Si vias from the structure of a MEMS wafer on a LSI wafer. The wafer level transfer methods are categorized to film transfer, device transfer connectivity last, and immediate connectivity at device transfer. Applications of these transfer methods are film bulk acoustic resonator (FBAR) on LSI, lead zirconate titanate (Pb(Zr,Ti)O_3_) (PZT) MEMS switch on LSI, and surface acoustic wave (SAW) resonators on LSI using respective methods. A selective transfer process was developed for multiple SAW filters on LSI. Tactile sensors and active matrix electron emitters for massive parallel electron beam lithography were developed using the through-Si vias.

## 1. Introduction

**Figure 1:**
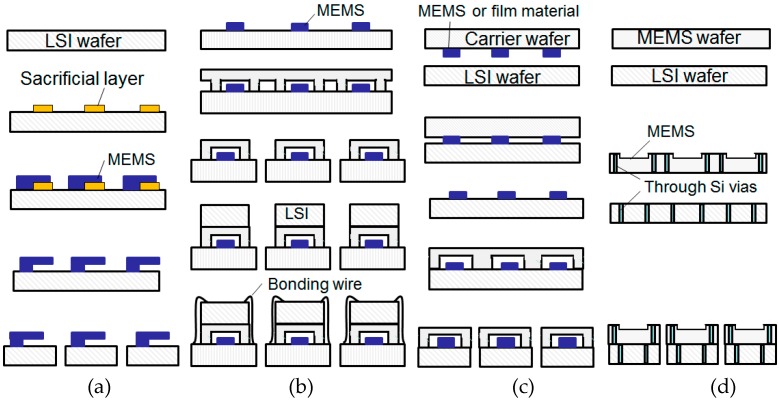
Heterogeneous integration processes; (**a**) surface micromachining; (**b**) assembly of packaged MEMS and LSI; (**c**) wafer-level transfer of MEMS to LSI (combined with a wafer level packaging); and (**d**) stacked integration using through Si vias.

MEMS—microelectromechanical systems—play important roles as key components for sensors. They are made on a Si wafer using microfabrication based on photolithography. MEMS are being used combined with integrated circuit (IC) or large scale integration (LSI) in different ways. Our purpose is to advance such combinations (heterogeneous integration) to make them suitable for various applications. Figure 1 shows four wafer processes for the heterogeneous integration: (a) surface micromachining; (b) assembly of packaged MEMS with LSI; (c) wafer-level transfer of MEMS to LSI; and (d) stacked integration using through-Si vias (TSVs). Figure 1c,d are the stacked integration methods focused on in this paper.

Figure 1a shows the surface micromachining that uses a sacrificial layer, which is etched out later, to make MEMS structures on a LSI wafer. The surface micromachining is suitable for making array MEMS, such as mirror arrays for displays and infrared imagers, because high density interconnection between the MEMS and the LSI can be achieved owing to the monolithic integration. However the MEMS fabrication has to be carried out on the LSI wafer. The applicable temperatures for the MEMS fabrication will, therefore, be limited to those acceptable for the LSI. 

Figure 1b shows the assembly of packaged MEMS and LSI that uses LSI chips located on or beside the packaged MEMS chips. The interconnections between them are made by wire bonding or flip-chip assembly. Since the MEMS are packaged, conventional plastic molding can be applied to protect the assembled devices. The MEMS fabrication process has flexibilities; however, the number of the interconnections is limited. The presence of stray capacitance and parasitic inductance because of the bonding pads and wires makes radio frequency (RF) applications difficult. Such a process is used for capacitive sensors, e.g., accelerometers and gyroscopes. 

**Figure 2:**
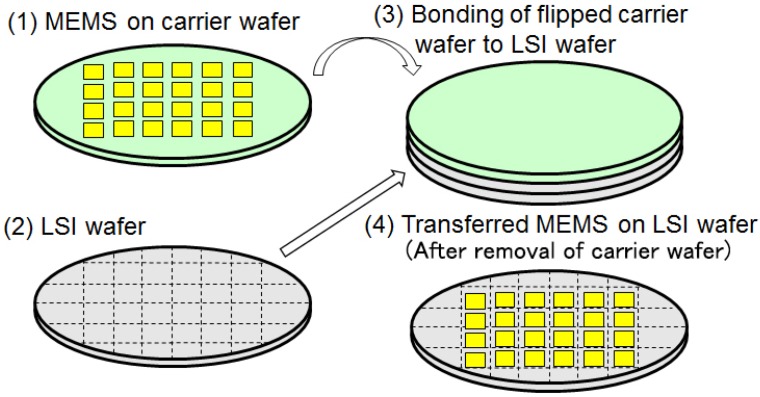
Concept of the heterogeneous integration by the wafer-level transfer.

Figure 1c demonstrates that the wafer-level transfer of MEMS to LSI enables advanced heterogeneous integration of MEMS on LSI [1]. MEMS or film of functional materials as lead zirconate titanate (Pb(Zr,Ti)O_3_) (PZT) are fabricated on a carrier wafer. They are transferred on a LSI wafer by bonding and by removing the carrier wafer. Wafer level packaging [2] can also be applied as shown in this process. The MEMS on LSI wafer is diced after being covered with a lid wafer. The concept of the heterogeneous integration by the wafer level transfer is schematically shown in Figure 2. MEMS on a carrier wafer (1) and a LSI wafer (2) are prepared. The carrier wafer is flipped and bonded to the LSI wafer in (3). By removing the carrier wafer, transferred MEMS on the LSI wafer (4) are obtained. The heterogeneous integration by the wafer level transfer of MEMS to LSI will be described in Section 2, with corresponding applications.

The Figure 1d is the process sequence of the stacked integration using the TSVs. Although both the MEMS wafer and the LSI wafer have TSVs in this example, there can be variations. The stacked integration using the TSVs will be described in Section 3 with applications for a tactile sensor network and active matrix electron emitters.

## 2. Wafer-Level Transfer of MEMS to LSI

**Figure 3:**
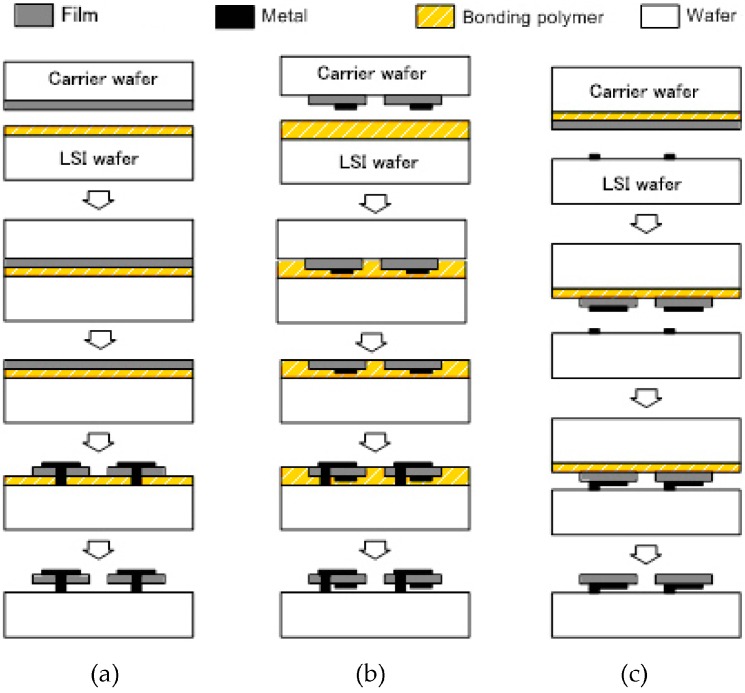
Wafer level transfer of heterogeneous MEMS to LSI; (**a**) film transfer; (**b**) device transfer connectivity last; and (**c**) immediate connectivity at device transfer.

The wafer level transfer of MEMS or film is carried out by three different methods as shown in Figure 3 and these are (a) film transfer; (b) device transfer connectivity last and (c) immediate connectivity at device transfer [3]. Wafer-to-wafer alignment plays important roles for the wafer level transfer especially for the processes shown in Figure 3b,c [4]. 

In Figure 3a, a film such as PZT is formed on a carrier wafer by deposition or by other methods. The film on the carrier wafer is bonded to a LSI wafer using bonding polymer (adhesive) and the carrier wafer is etched out. MEMS are fabricated using the film and then electrical and mechanical connections to the LSI wafer are made by a deposited metal. The MEMS on LSI can be obtained by etching out the bonding polymer. Since the MEMS are fabricated on the LSI wafer, the process is controlled to prevent damage to the LSI. The film transfer technique has been used for producing LED printer heads [5]. In this case, a double-heterostructure AlGaAs/GaAs/AlGaAs for LEDs is transferred on a LSI wafer directly by Van der Waals bonding without polymer.

The process sequence of the device transfer connectivity last is shown in Figure 3b. MEMS are fabricated on a carrier wafer. The MEMS wafer is bonded to a LSI wafer using the bonding polymer. The carrier wafer is etched out and the electrical and mechanical connections to the LSI wafer are made by a deposited metal. The MEMS on LSI can be obtained by etching out the bonding polymer. This process is called connectivity last because connections are made after bonding.

Figure 3c is the process sequence of the immediate connectivity at device transfer. Film for MEMS is bonded to the carrier wafer with a polymer. Bumps for electrical and mechanical connection are formed on the LSI wafer. MEMS are fabricated on the carrier wafer using the film and the MEMS wafer is bonded to the LSI wafer with the bumps. Finally the carrier wafer is removed by etching out the bonding polymer and we can get the MEMS on LSI. Since the connections are made at the device transfer, this process is called immediate connectivity at device transfer. A similar process has been used, i.e., for a back-illuminated stacked image sensors which have an image sensor LSI on a logic LSI made by wafer-level bump bonding [6]. Atomic force microscope (AFM)-based data-storage systems also use the immediate connectivity at device transfer [7].

### 2.1. Film Transfer (Film Bulk Acoustic Resonator on LSI)

**Figure 4:**
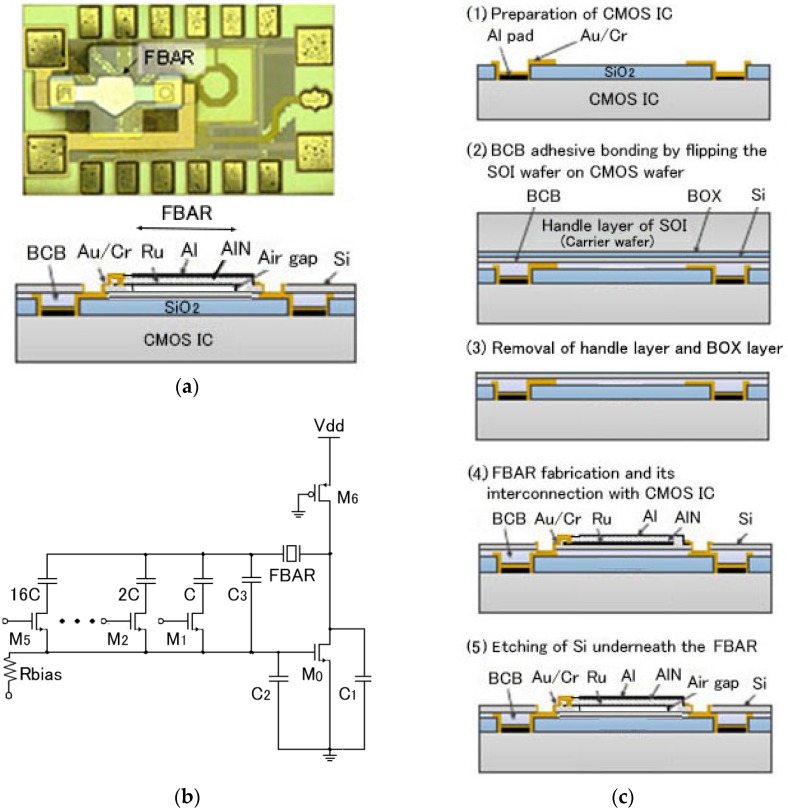
FBAR on LSI; (**a**) photograph and structure; (**b**) CMOS oscillation circuit; and (**c**) fabrication process.

Film bulk acoustic resonator (FBAR) on 0.18 μm complementary metal oxide semiconductor (CMOS) LSI was developed for voltage controlled oscillator (VCO) of 2.45 GHz [8]. The photograph and the structure are shown in Figure 4a. The FBAR is composed of a free-standing piezoelectric film (1.76 μm thick *c*-axis AlN) with a bottom electrode (100 nm thick Ru) and a top electrode (100 nm thick Al). AlN was chosen for its high bulk acoustic velocity for high frequency and low deposition temperature (300 °C) for CMOS compatibility. The AlN was deposited by a reactive sputtering in nitrogen environment using Al as a target. The CMOS oscillation circuit for the VCO is shown in Figure 4b. The fabrication process (Figure 4c) is as follows: CMOS IC is prepared (1) and is bonded to a silicon-on-insulator (SOI) wafer with a bonding polymer benzocyclobutene (BCB) (2) and a 50 μm thick BCB from Dow Co. was spin coated. The bonding was performed at 270 °C with 1.25 MPa bonding pressure for 10 min. The SOI wafer is composed of a thin Si active layer on a buried oxide (BOX) layer supported on a thick Si handle layer. The handle layer corresponds to the carrier wafer and the thin Si layer corresponds to the film in Figure 3a. The Si handle layer is removed by reactive ion etching (RIE) using SF_6_ as an etching gas or by grinding/polishing. By wet etching the BOX layer in buffered HF, the thin Si layer is transferred on the LSI wafer (3). The bottom Ru electrode is sputter-deposited and patterned. The AlN is also sputter-deposited and patterned. The top Al is fabricated using a lift-off process. After RIE of Si thin film and the BCB, Cr and Au patterns are formed using the lift-off process for metal interconnection (4). The thin Si layer underneath the FBAR is etched out using a XeF_2_ gas. Finally the photoresist mask used to cover the surface except the FBAR area is removed by O_2_ plasma etching (5). This process is modified from the film transfer (Figure 3a) as follows: the AlN layer was deposited on the transferred film and the bonding polymer was not removed. Like this, there can be different variations in the process. CMOS-based amperometric sensor array was fabricated by transfer of boron-doped diamond film deposited on a carrier wafer at 800 °C [9].

### 2.2. Device Transfer Connectivity Last (PZT MEMS Switch on LSI)

**Figure 5:**
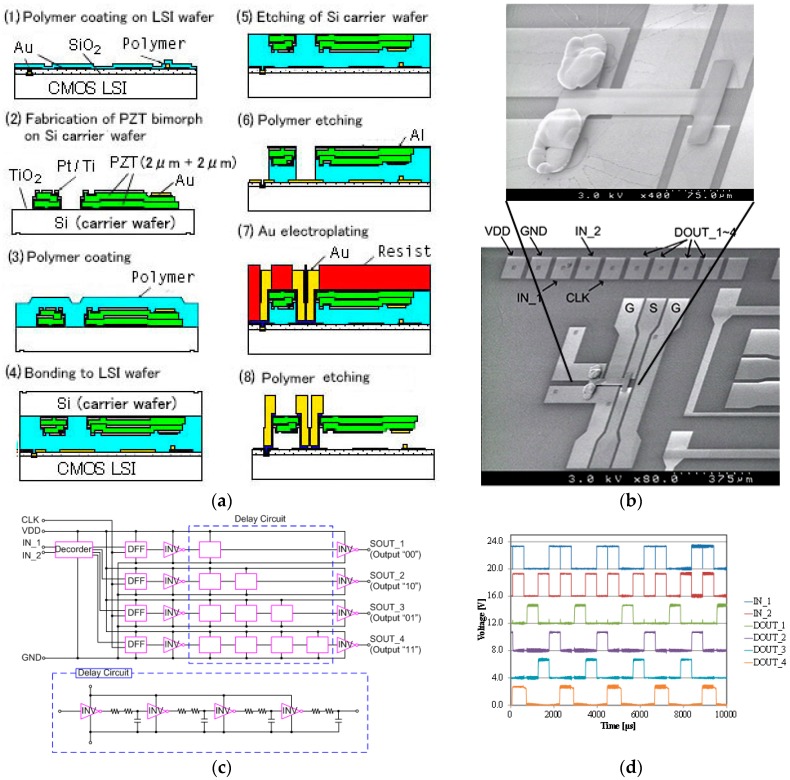
PZT MEMS switch on LSI; (**a**) fabrication process; (**b**) photographs; (**c**) circuit; and (**d**) waveform from the circuit.

PZT-actuated MEMS switches were fabricated on a 0.35 μm CMOS LSI wafer using the device transfer connectivity last method [10]. The fabrication process and photographs are shown in Figure 5a,b respectively. The piezoelectric MEMS switch works at lower driving voltage and occupies a smaller area than electrostatic MEMS switches. The piezoelectric MEMS also enable a wide range of variable MEMS capacitors owing to their no pull-in phenomena compared to electrostatic variable capacitors. The fabrication process (Figure 5a) is as follows: PZT is deposited on a Si carrier wafer by a sol-gel method. A sol-gel solution is spin-coated repeatedly with annealing at 680 °C in O_2_. A bimorph structure made of two 2 μm thick PZT layers with Ti and Pt metallization is formed symmetrically in order to prevent bending due to intrinsic and thermal stresses (2). The flipped Si carrier wafer is bonded to the LSI wafer using aromatic compounds polymer (NST1029 Nissan Chemical Industries, Ltd., Tokyo, Japan) (3, 4). The bonding was performed at 150 °C with 1MPa bonding pressure for 60 min. The PZT layers are transferred to the LSI wafer by etching out the Si carrier wafer (5). The MEMS and the LSI are connected using an electroplated metal (6, 7). Finally, the polymer is removed by O_2_ plasma to release the MEMS switches (8). The deflection of the PZT bimorph beam obtained was 5 μm at 8 V. The evidence of the circuit function after finishing the fabrication process is shown in Figure 5c,d. The circuit of the LSI was not damaged during the fabrication process and the operation of the switch control circuit was confirmed.

### 2.3. Immediate Connectivity at Device Transfer (Surface Acoustic Wave Resonator on LSI)

**Figure 6:**
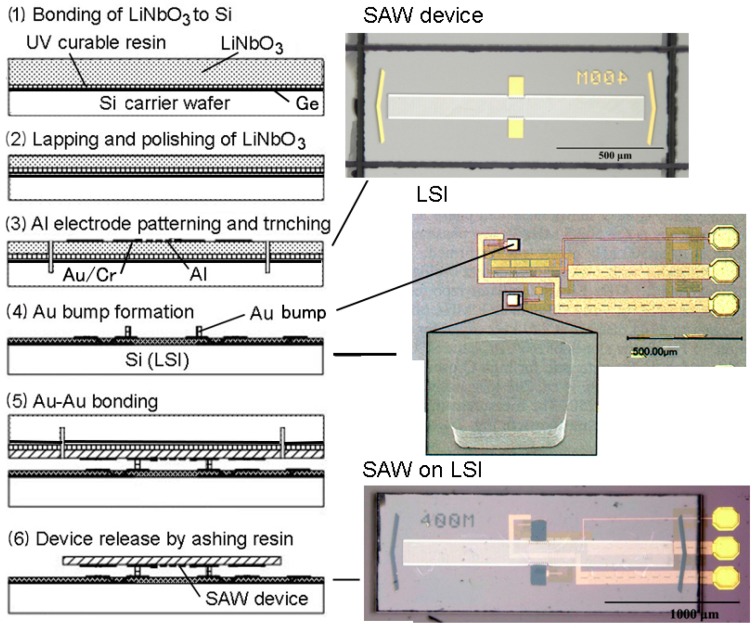
Fabrication process of SAW resonator on LSI.

A lithium-niobate (LiNbO_3_)-based surface acoustic wave (SAW) resonator was heterogeneously integrated by the immediate connectivity at device transfer method [11]. The fabrication process of the SAW resonator on LSI is shown in Figure 6. A LiNbO_3_ wafer is temporary bonded to a Ge layer on the Si carrier wafer by UV curable resin (1). The LiNbO_3_ wafer is thinned by lapping and polishing (2). Al electrodes for the inter-digital transducer (IDT) for the SAW resonator and Au on Cr for bonding are formed by deposition and patterning. Trenches are made in the LiNbO_3_ wafer and the Si carrier wafer by dicing (3). Au bumps are formed on the LSI wafer by Au electroplating using 20 μm thick photoresist as a mask. The surface of the Au is made flat by single-point diamond cutting lathe (4). The flat-finished Au surface is shown in the photograph of the LSI in Figure 6. The flipped SAW device on the Si carrier wafer is bonded to the two bumps using Au-Au bonding (5). Plasma treatment to activate the Au surface is applied to lower the bonding temperature to 150 °C in order to overcome the large thermal expansion mismatch between the LiNbO_3_ (15 ppm/K along the *α*-axis) and Si (2.6 ppm/K). The treatment was carried out in Ar plasma using homemade equipment. The Ge layer is etched out in H_2_O_2_ to remove the Si carrier wafer. A 500 MHz one-chip SAW oscillator was prototyped. A low phase noise of −122 dBc/Hz at 10 KHz offset and −160 dBc/Hz at 500 kHz offset was achieved owing to the low stray capacitance and inductance.

**Figure 7:**
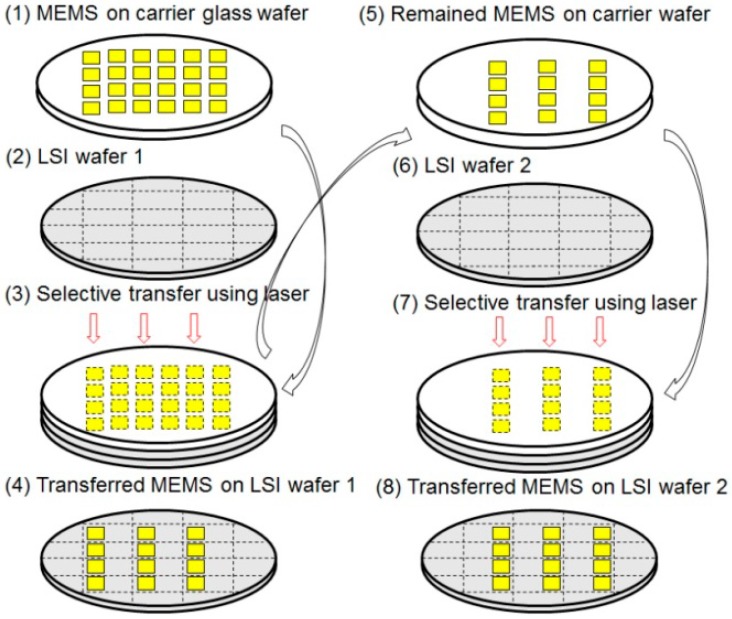
Concept of selective transfer.

Wafer level selective transfer from one carrier wafer to multiple LSI wafers is needed for cost-effective integration of dies with different sizes [12]. The concept of the selective transfer is shown in Figure 7. The MEMS wafer is bonded to a glass carrier wafer using a polymer (acrylic resin) and grooves are made on the MEMS wafer by dicing (1). The MEMS wafer on the carrier wafer is aligned with the LSI wafer 1 and bonded by the Au-Au bonding using bumps as explained in Figure 6 (5). Selective de-bonding is made by irradiating the interfacial acrylic resin using a Nd:YVO_4_ third harmonic laser (λ: 355 nm) through the glass carrier wafer (3). The acrylic resin is carbonized to lose adhesion and the MEMS devices are transferred to the surface of the LSI wafer 1 (4). The MEMS dies remaining on the glass carrier wafer can be transferred to another LSI wafer (LSI wafer 2) (5–8).

**Figure 8:**
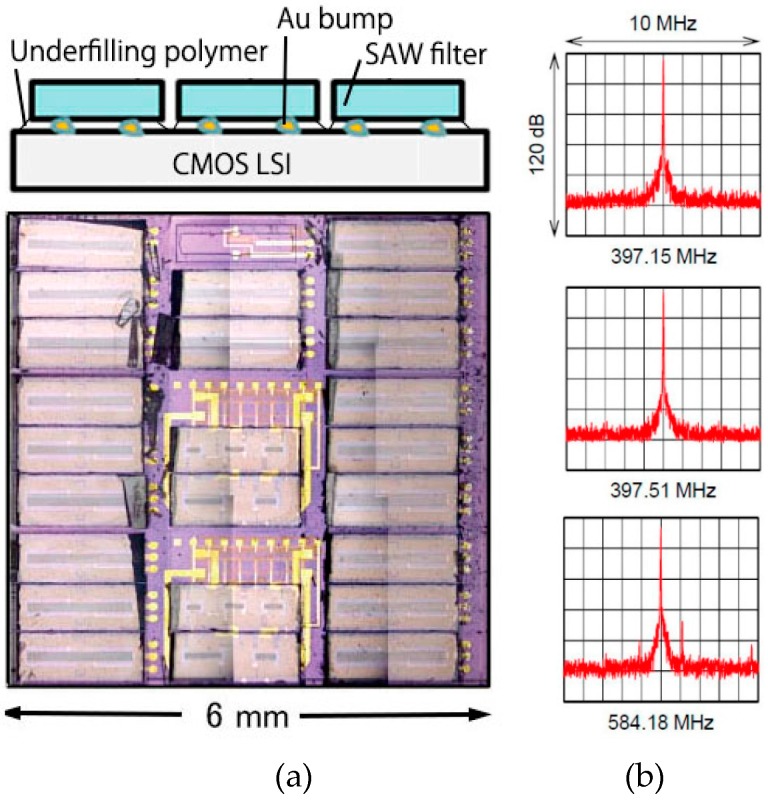
Multi SAW resonators on a LSI chip; (**a**) structure and photograph; and (**b**) frequency spectrums of three SAW oscillators on LSI.

Multiple SAW resonators were formed on a LSI chip as shown in Figure 8a [13]. The selective transfer method explained in Figure 7 was applied from multiple carrier wafers to multiple LSI wafers. The SAW resonators fabricated on a LiNbO_3_ wafer is bonded to a glass carrier wafer using the polymer. After the Au-Au bonding to the LSI wafer using bumps, selective laser de-bonding is applied and, finally, polymer underfilling fixes the SAW chips on the LSI chip. Frequency spectra of three SAW oscillators on the LSI chip are shown in Figure 8b.

## 3. Stacked Integration Using through-Si Vias

**Figure 9:**
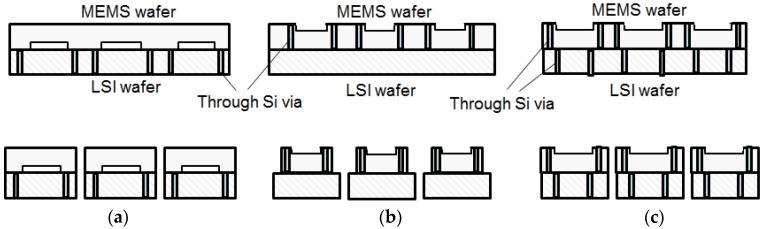
Stacked integration using through Si vias (TSVs); (**a**) LSI wafer vias; (**b**) MEMS wafer vias; and (**c**) LSI wafer vias and MEMS wafer vias.

Through-Si vias (TSVs) can be used for stacking and connecting MEMS wafer and LSI wafers [14]. Figure 9 shows three different methods: (a) LSI wafer vias; (b) MEMS wafer vias; (c) LSI wafer vias and MEMS wafer vias.

### 3.1. LSI Wafer Vias (Tactile Sensor Network)

**Figure 10:**
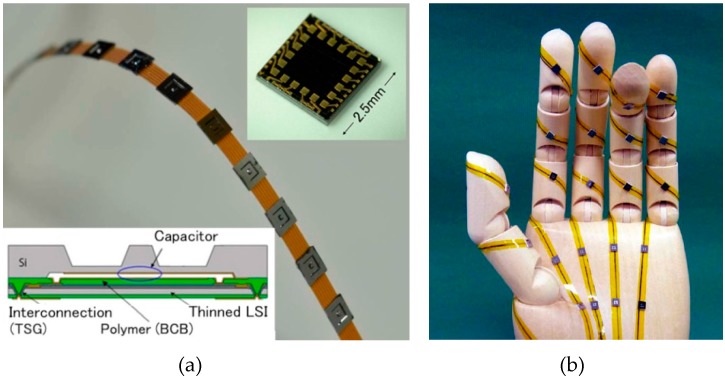
Tactile sensor network; (**a**) structure and photograph; and (**b**) application.

Distributed tactile sensors (tactile sensor network) are needed on the skin of safe nursing care robots, rehabilitation robots, etc. to ensure their collision safety and to enable body communication. The structure and the photograph of the tactile sensor network are shown in Figure 10a [15]. A capacitive tactile force sensor is formed on a communication LSI by adhesive bonding using the BCB. The force is detected as a capacitance change and it is converted to a digital output. Tactile sensor chips are connected to a flexible cable which has common four-wire bus for power supply, ground and two signals. The interconnection pads to the flexible cable are made on the backside of the communication LSI using the LSI wafer vias (Figure 9a called through-silicon groove (TSG). Figure 10b is an example of the application of the tactile sensor network.

**Figure 11:**
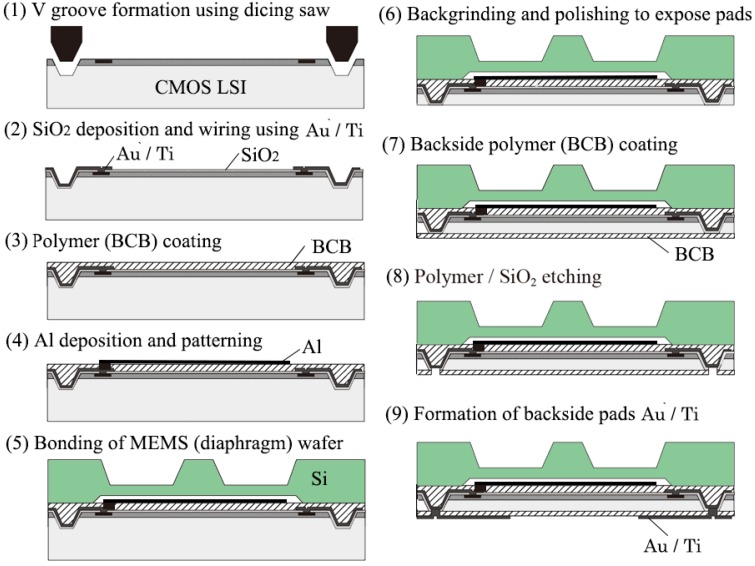
Fabrication process of the tactile force sensor on LSI.

**Figure 12:**
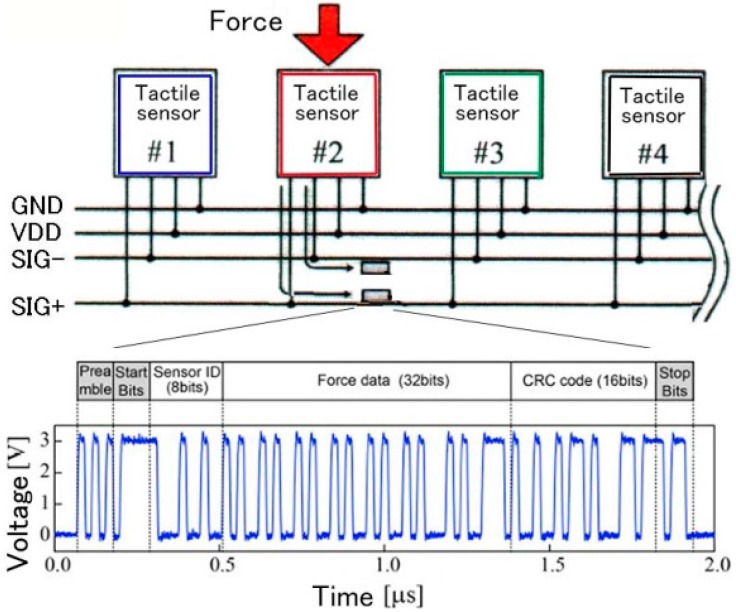
Packet communication for the tactile sensor network.

The fabrication process of the tactile sensor is shown in Figure 11 [16]. V grooves are formed on the LSI wafer using a dicing saw (1). The groves are insulated by depositing SiO_2_ (2) and then metal interconnections are made between boding pads and the grooves by Ti and Au (3). Polymer (BCB) is coated (4) and the Al pattern is made for the sensing capacitor after making vias in the polymer (5). Si MEMS wafers having diaphragms for sensing capacitors are bonded to the polymer (6). The BCB is half-cured before the Al deposition at 225 °C for 30 min and patterning and it was full-baked after bonding [16]. The LSI wafer is thinned from the backside by grinding and polishing. This exposes the bottom of the V groove (7). BCB is coated on the back side (8) and the BCB and the SiO_2_ are etched to expose the metal connected from the front side (9). Finally, pads on the backside are formed by Ti and Au (10). The function of the communication LSI for event driven data transmission at a clock rate of 45 MHz was confirmed. An example of packet communication of the tactile sensor network is shown in Figure 12. Being event driven, the host computer recognizes the sensor position and the applied force from the sensor ID and the force data in the packet signal, respectively. 

### 3.2. MEMS Wafer Vias (Active Matrix Planar-Type Electron Sources for Massive Parallel Electron Beam Lithography)

Digital fabrication of LSI based on maskless lithography is expected for cost effective production and short development time. However extremely-high throughput direct electron beam (EB) lithography is required because the latest LSI wafer has up to 10^12^ (1 tera) nanoscale transistors on it. Massive parallel EB exposure systems with an active matrix nanocrystal Si (nc-Si) electron emitter are under development. The nc-Si is formed by anodizing Si in HF(55%) + C_2_H_5_OH (1:1) solution, followed by electrochemical oxidation (ECO) in ethylene glycol and potassium nitrate, high-pressure water vapor annealing (HWA), super-critical rinsing and drying (SCRD), and annealing in H_2_.

**Figure 13:**
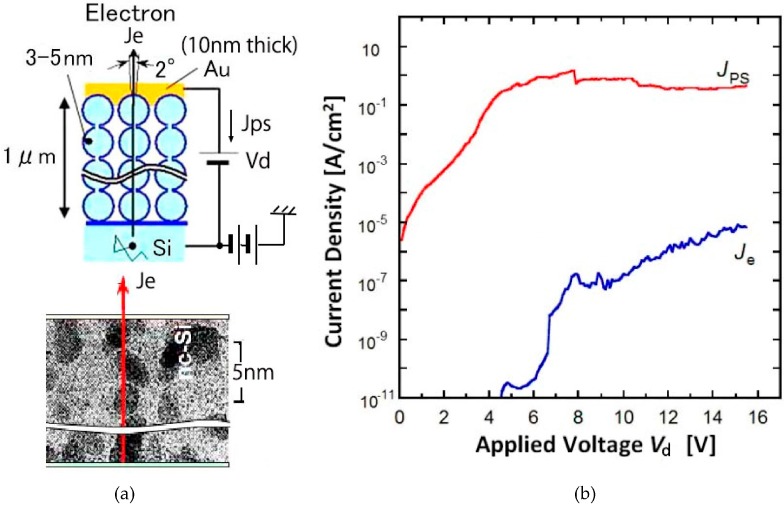
nc-Si electron emitter; (**a**) principle; and (**b**) characteristics.

The principle and the characteristics of the nc-Si emitter are shown in Figure 13a,b [17]. The nc-Si emitter consists of cascaded tunnel junctions and accelerated ballistic electrons are emitted through a thin (10 nm thick) Au layer. The emission current (Je) and the current to the thin Au layer (Jps) are plotted versus applied voltage (*V*_d_) in Figure 13b. The emitted current by the ballistic electrons is obtained at low voltage around 10 V. The low voltage electron emission is needed for making high-density, arrayed, active-matrix circuits because the size of the transistor depends on the voltage required. The active matrix LSI developed for 100 × 100 array is 100 μm pitch on a 10 mm square chip.

**Figure 14:**
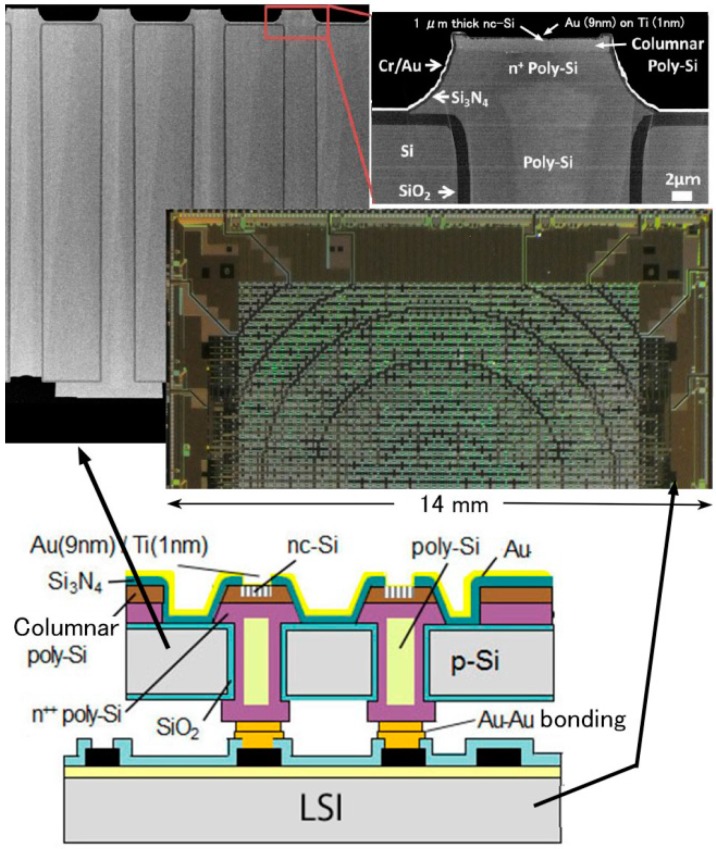
Structure of planer-type active matrix electron source using the nc-Si electron emitter.

**Figure 15:**
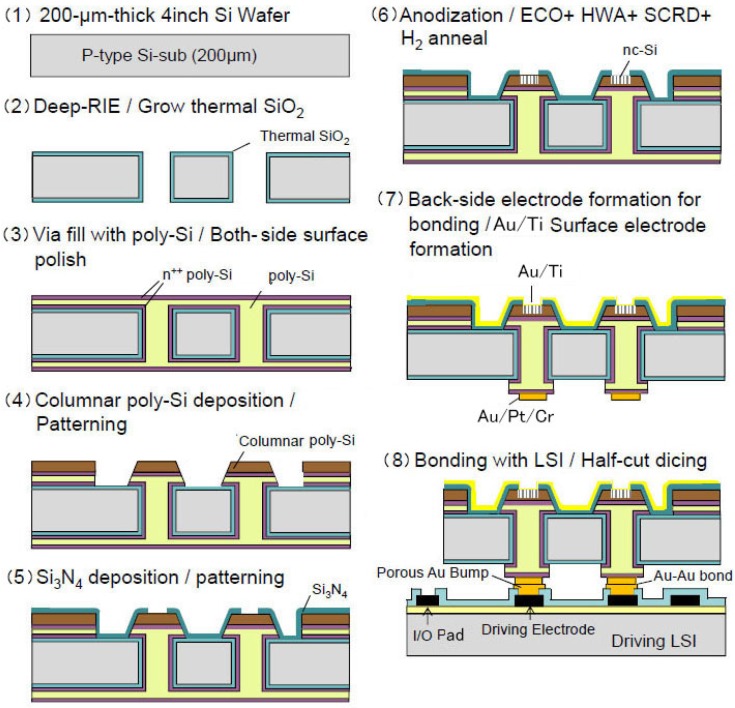
Fabrication process of planer-type active matrix electron source.

A planer-type nc-Si emitter array with TSVs was fabricated on LSI. The structure, the photograph of the cross-section of the TSVs which has nc-Si electron emitter on the top and the photograph of half of the LSI chip are shown in Figure 14 [18]. The structure was fabricated using the MEMS wafer vias (Figure 9b) as shown in Figure 15. The Si wafer is thermally oxidized after deep RIE; reactive ion etching to make through holes (2). The vias are formed by poly-Si deposition and planarization (polishing) (3). Columnar poly-Si is deposited for the purpose of the nc-Si and then poly-Si is patterned (4). Si_3_N_4_ is deposited and patterned (5). The next process step is the formation of the nc-Si. The poly-Si is anodized and treated by the method described above (6). Back-side electrodes for the bump bonding and the thin surface electrode (Au (9 nm) on Ti (1 nm)) are formed (7). Finally the wafer is bonded to the LSI wafer with porous Au bumps (Au-Au bonding) [19] and I/O pads of the LSI is exposed by half-cut dicing (8). 

**Figure 16:**
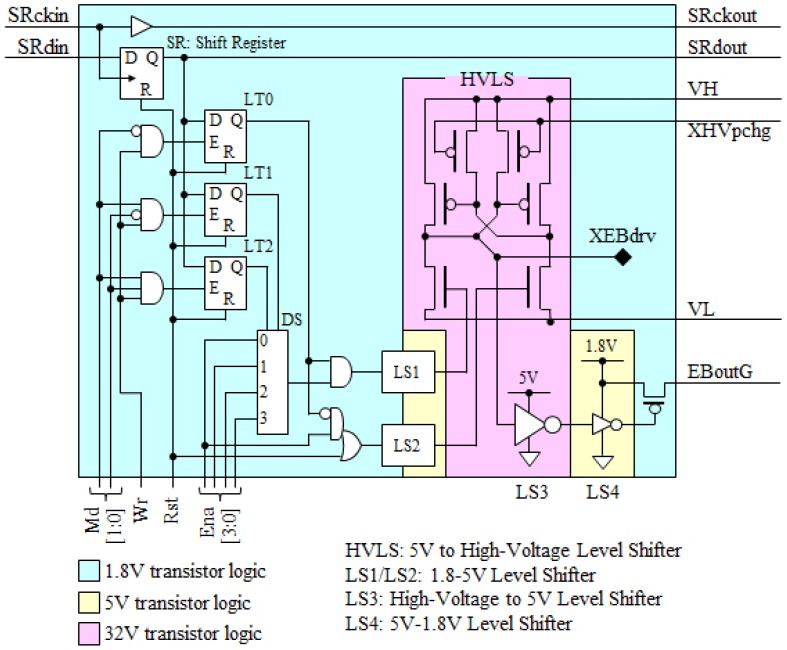
Circuit of one cell in the 100 × 100 active matrix driving LSI.

The circuit of one cell in the 100 × 100 active matrix driving LSI is shown in Figure 16 [18]. The core region of the chip is 10 × 10 mm^2^ and each cells are laid out with a 100 μm pitch to match that of the electron emitter as shown in Figure 14. The LSI was fabricated through a 0.18 μm CMOS high-voltage process that involves 1.8 V/6 V/32 V transistors in the chip. The chip has an innovative aberration correction scheme using dielectric ring isolation as shown in the chip photograph in Figure 14. It has also TSVs for electrical interconnection on the back side of the chip. A compensation scheme for electron beam intensity variation and a test scheme are involved in each cell. The bitmap data for the active matrix control of each emitter is shifted in the chip at 100 MHz and can update the 100 × 100 data within 1 μs. The basic function of the fabricated LSI was confirmed. 

### 3.3. LSI Wafer Vias and MEMS Wafer Vias (Active Matrix Pierce-Type Electron Sources for Massive Parallel Electron Beam Lithography)

**Figure 17:**
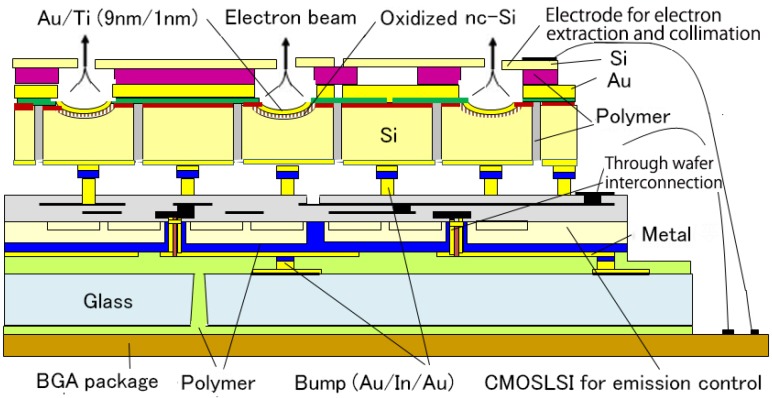
Schematic of a Pierce-type nc-Si electron emitter array stacked with a driving LSI.

**Figure 18:**
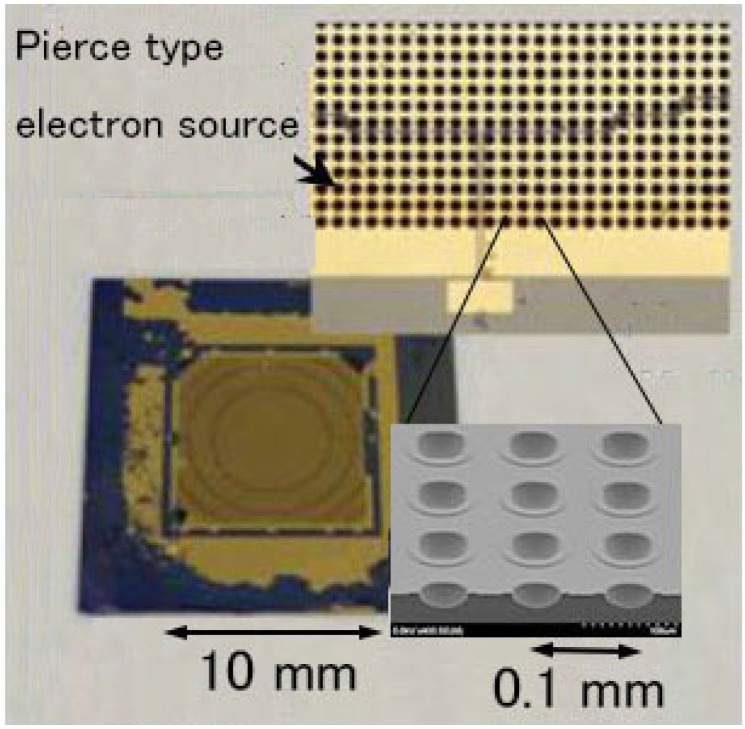
Photographs of Pierce-type nc-Si electron emitter array.

The other nc-Si electron emitter array, called a Pierce-type, on the driving LSI is shown in Figure 17 [20]. The emitter array consists of 100 × 100 hemispherical emitters formed by isotropic wet etching of Si. The nc-Si is fabricated on the surface of the concave hemispherical surface. The electron beam can be condensed and collimated by using an extraction electrode bonded on the surface. Trenches are made by the deep RIE and filled with polymer (BCB) for the purpose of the dielectric isolation. The emitter chip is bonded to the driving LSI supported on a glass plate using Au-In bumps. The photograph of the Pierce-type emitter array, which lacks the extraction electrode, is shown in Figure 18.

**Figure 19:**
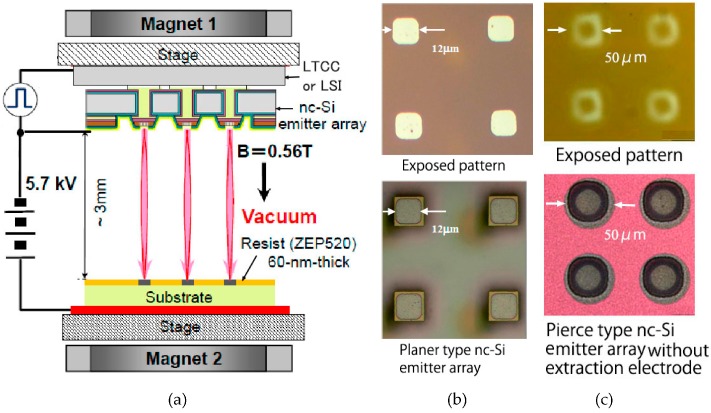
1:1 electron beam exposure; (**a**) exposure system; (**b**) planer type; and (**c**) Pierce type.

Although the nc-Si electron emitter arrays (planer type and Pierce type) and the LSI are fabricated successfully, the fabrication of the stacked structures have not been succeeded yet. Pattern transfer experiments by electron beam exposure were made [18]. The 1:1 exposure setup is shown in Figure 19a. The emitted electrons are accelerated at 5.7 kV and are directed onto a target substrate placed 3 mm away. The nc-Si emitters are driven by 14 V pulse with 250 ms duration. A 60 nm thick ZEP520 EB resist was exposed until it reaches a total electron dose of 30 μC·cm^−2^. A magnetic field of 0.56 T in parallel with the electric field was applied using two permanent magnets. This causes the emitted electrons to spiral around the magnetic field lines and to focus on the target. The pattern images exposed by the planer-type and the Pierce-type without an extraction electrode are shown in Figure 19b,c respectively together with the images of the emitter surfaces. Patterns of the emitters were reproduced on the EB resist. 

## 4. Conclusions 

Stacked integration by wafer-level transfer of a device/film on a carrier wafer to a LSI wafer and by stacking and connecting the MEMS wafer and the LSI wafer using through-Si vias (TSVs) are described. 

The integration processes based on the wafer-level device/film transfer are carried out at low temperature to prevent damage to the LSI. The wafer-level transfer has great advantages in contrast to the assembly-level integration as follows: not only can we reduce the chip size and the cost, but we can also increase the number of the MEMS elements and interconnections between the MEMS and the LSI; additionally, we can reduce the stray capacitance and the stray inductance for high performance capacitive sensors and RF devices; functional materials, such as PZT, can be used by the film transfer; different sized MEMS from LSI can be stacked by the selective transfer. As examples of the wafer-level stacked MEMS and LSI using TSVs, the tactile sensor network and the active matrix electron emitters for maskless electron beam lithography are described. These stacked integrations can achieve advanced microsystems needed in different applications.
